# Factors Associated with Support for Adolescent Access to Contraception Among Mexican Catholic Parents

**DOI:** 10.1007/s10943-021-01186-w

**Published:** 2021-02-06

**Authors:** Stephanie A. Küng, Biani Saavedra-Avendano, Evelyn Aldaz Vélez, María Consuelo Mejía Piñeros, Gillian M. Fawcett Metcalfe, Blair G. Darney

**Affiliations:** 1 Ipas, Chapel Hill, NC USA; 2 Centro de Investigación y Docencia Económicas, Mexico City, Mexico; 3 Católicas por el Derecho a Decidir, Mexico City, Mexico; 4 Department of Obstetrics and Gynecology, Oregon Health and Science University, Portland, OR USA; 5 OHSU-PSU School of Public Health, Portland, OR USA; 6 Instituto Nacional de Salud Pública (INSP), Centro de Investigacion en Salud Poblacional (CISP), Cuernavaca, Mexico

**Keywords:** Latin America and the Caribbean, Catholicism, Contraception/family planning, Adolescents, Measuring religiosity

## Abstract

We used a nationally representative survey of 2186 Mexican Catholic parents to assess two outcomes: support for adolescent access to modern contraception and whether adolescents unaccompanied by an adult should have access to contraceptive methods. A majority (85%) of Mexican Catholic parents support adolescent access to modern contraceptive methods, but there was less support (28%) for access to contraception unaccompanied. Further, our results show strong support (92%) for sex education in schools. Parents who believe that good Catholics can use contraception had higher odds of support for adolescent access and unaccompanied access to modern contraception. Mexican Catholic parents support adolescent access to modern contraception, but support for unaccompanied access to contraception is lower. This may reflect an interest in being involved, and not necessarily opposition to contraceptive use. Measures of Catholicism that focus on behaviors may better explain opinions about adolescent access to contraception.

## Introduction

In Mexico, access to family planning is established as a right of Mexican citizens and national policy states that adolescents have access to all modern methods of contraception (Ley General de Salud 1984; Consejo Nacional de Población 2014). Despite progressive policy, studies show that unmet need for contraception is higher among younger women in Mexico than among older women, especially among young women living outside of Mexico City (Juarez et al. 2018), and is increasing among certain adolescent subpopulations, including married adolescents and those living in rural areas (Juarez et al. 2010). Adolescent pregnancy rates remain high in Mexico, among the highest of OECD countries at 130 pregnancies per 1000 adolescents age 15–19 (OECD Family Database).

While many factors, including insurance, place of residence, education, and poverty, have been shown to affect adolescent access to contraception in Mexico (Juarez et al. 2018), parents and parental notification as barriers to contraceptive access among adolescents has also been documented (Dansereau et al. 2017; Reddy et al. 2002). Mexico is a very Catholic country; 77–85% of adults self-identify as Catholic (Católicas por el Derecho a Decidir 2014; National Institute of Statistics and Geography 2010), which is often viewed as a deterrent to access to sexual and reproductive health services. However, Mexican law is clear: NOM-005 states that family planning, including information and education, counseling, prescription and application, should be offered to all people of reproductive age, including adolescents, and that the consent of a parent is *not* required (Norma Oficial Mexicana NOM-005-SSA2-1993).

The purpose of this study is to identify factors associated with parental support for adolescent access to contraception among Catholic parents. We examine general support for adolescent access to modern contraceptives (support in theory) and support for access to contraceptive services without an adult (support in practice). While research has shown high support for sex education and contraceptive access among different religious groups in the United States (Constantine et al. 2007; Barrett et al. 2014), to our knowledge no literature exists examining factors associated with support for adolescent access to birth control among Mexican Catholic parents.

## Methods

We conducted a cross-sectional analysis using a nationally representative Spanish language survey collected in-person between July and September 2014 from self-identified Mexican Catholics over the age of 18 (Insad, *Report of the National Survey of Catholic Opinion)*. The Mexican Survey of Catholic Opinion (Católicas por el Derecho a Decidir 2014) includes information on household sociodemographics and a variety of topics, including sexual and reproductive health. Individuals were selected by multistage random sampling using Basic Geographic Areas which divide Mexico into 60,000 distinct geographic areas (Católicas por el Derecho a Decidir 2014). Within areas, random sampling was used to select households and determine eligibility (Catholic; over 18); the member of the household whose birthdate was nearest to the survey date completed the survey. The response rate among eligible households was 85%. Our study focuses on parents, so we excluded 483 respondents who reported not having any children for a final analytic sample of 2186 respondents.

We have two binary outcomes, support for adolescent access to modern contraception in theory and practice. Respondents were first asked a question about whether or not they believe adolescents should have access to all modern contraceptive methods, defined in the question as pills, injectables, IUDs, and implants. We label this access in theory. Respondents were then asked if, for doctors to give these methods to adolescents, adolescents should be accompanied by an adult or should be able to go on their own. We label this outcome access in practice as it measures parents’ support for the actual operationalization of access to contraception without the presence or consent of an adult.

We examined several measures of the degree of Catholicism, Catholic beliefs, and values. We included two measures of religiosity: self-identified strength of Catholicism (very Catholic, somewhat Catholic, and not at all/a little Catholic) and perceptions of a good Catholic; particularly, a binary indicator of whether someone who uses contraception can continue to be a good Catholic. We selected these measures of religiosity based on previous work (Küng et al. 2018), which found that self-reported strength of Catholicism and behaviors of “good” Catholics had more explanatory power than attendance at mass or other measures. We explored additional measures about religion and spirituality: respondents were asked about their idea of God, which we dichotomized into God as protecting vs. forgiving, punishing and rewarding, or imposing rules. Respondents were also asked to identify what they believe is the most important Catholic value, dichotomized into respect vs. love, liberty, forgiveness, obedience, justice, or mercy. We chose reference categories that we believed may be associated with contraception acceptance; for example, that God is protective may translate into a belief that God protects from pregnancy or the value of respect as respecting personal choices about pregnancy and its prevention. Respondents also indicated whether they thought that the Catholic Church should permit Catholics to use contraception.

We included individual and household-level sociodemographics in our analysis. We included age, income, education, Mexico City residence, number of children, and respondent’s current contraceptive use. We grouped age into four categories: 18–30, 31–40, 41–50, and 51 + . We used irregular age bands to account for the smaller proportion of parents in the 18–30 range. Income represents the range of monthly income in pesos, converted to USD (using the September 30, 2014 exchange rate): < $145, $145–434, $435–723, and $724 + . In 2014, minimum wage in Mexico ranged between 63.77 and 67.29 pesos a day depending on geography (Secretaria de Trabajo y Previsión Social 2014), amounting to a minimum monthly wage of 1913 to 2019 pesos (142–150 USD). We divided our education variable into 3 categories: less than primary school completed or no schooling, secondary school (9th grade) completed, and high school or more advanced schooling. Where we describe Mexico City residence, we dichotomized between respondents who live within and outside Mexico City, since previous literature shows increased access to birth control within Mexico City (Juarez et al. 2010). We dichotomized number of children into 1–2 and more than three children. We included current use of any method of contraception (respondent or respondent’s partner) and support for sex education in public schools. Finally, we included an indicator of opinion about why adolescents get pregnant, which was an open-ended question that we dichotomized into believing adolescent pregnancy is due to factors outside of the adolescent’s control (i.e., adolescents get pregnant because of issues outside of their control—lack of information or communication, family problems, rape) and believing adolescent pregnancy is within control of the adolescent (i.e., adolescents get pregnant because they like to rebel, for curiosity/experimentation, or because they are irresponsible in using birth control).

We applied survey weights to account for the complex survey design and allow inference to the national Catholic population. First, we characterized our sample. We then calculated overall agreement with our two outcomes. We used bivariate tests to examine the distribution of our outcomes by different measures of Catholicism (beliefs, values, religiosity). We also assessed the correlation between all Catholic measures and ran multivariable models with each Catholic measure individually and then together to assess how the inclusion or exclusion of different measures may affect findings. We then developed two logistic regression models, one for each outcome. We included covariates based on literature, results from bivariate tests, and our own previous work (Küng et al. 2018). We did not include all Catholic value and belief variables in our final models; we excluded the measures of idea of God and respect as the most important Catholic value due to nonsignificance in bivariate analysis. We excluded the belief that the Catholic Church should permit Catholics to use contraception due to the correlation with belief that a person who uses contraception can continue being a good Catholic. We opted for the variable measuring whether a person who uses contraception can continue being a good Catholic over the belief that the Catholic Church should permit Catholics to use contraception based on existing literature and theoretical importance of the good Catholic variable as a measure of Catholicism and perceptions and values around Catholicism rather than Church policy. We included only the measure of opinion that a person who uses contraception can continue being a good Catholic and the self-identified strength of Catholicism measure in our multivariable models. Also, in our final models, we controlled for support for public schools teaching sex education, opinion about why adolescents get pregnant, gender, age, income, Mexico City residence, use of contraception, and number of children.

We conducted several sensitivity analyses. We tested for collinearity between income and education and dropped each to assess impact on model estimates; there were no meaningful changes in odds ratios, and we retained both variables. We examined potentially nonsensical responses, such as the 44 respondents who said they support unaccompanied access to contraception but do not support access to modern methods; however, models were robust to the exclusion of these respondents, so we retained them in the analysis. We tested whether support for sex education moderated the relationship between our measures of Catholicism and support for adolescent access to contraception using an interaction term; it was not significant, and we include only fixed effects in the final model. We used the Bayesian Information Criterion (BIC) to compare models and select the final model (lowest BIC). All statistical analyses were conducted using STATA version 14.2. The OHSU Institutional Review Board deemed this secondary analysis not human subjects research.

## Results

Table 1 displays crude and weighted sample characteristics. Just over half the sample of parents was female (weighted 56%; 95% CI: 53–59%), and most (78%; 95% CI: 71–86%) were over the age of 30. A large majority of respondents (92%; 95% CI: 91–94%) lived outside of Mexico City. Just over half of respondents (52%; 95% CI: 49–55%) had more than three children and almost half (41%; 95% CI: 38–44%) reported having a primary education or no education. Just under two-thirds (62%; 95% CI: 59–65%) reported that either they or their partner currently use contraception. Nearly all of the sample (92%; 95% CI: 90–93) believed that public schools should teach sex education.

**Table 1:** Sample characteristics, Mexican Catholic Parents, 2014

	Crude *n*	Crude % *n* = 2186	Weighted % [95% CI] Weighted *n* = 61,332,862
Sex			
Female	1404	64	56 [53–59]
Age			
18 to 30	418	19	22 [19–25]
31 to 40	578	26	25 [23–28]
41 to 50	448	20	21 [19–24]
51 +	742	34	32 [29–34]
Education (*n* = 2185)			
None/primary	1032	47	41 [38–44]
Secondary	676	31	32 [29–34]
High school or more	477	22	28 [25–31]
Income, USD (*n* = 2172)			
< $145	983	45	38 [35–41]
$145–434	775	36	38 [35–41]
$435–723	288	13	16 [14–18]
≥ $724	126	6	8 [6–10]
Mexico city residence	228	10	8 [6–9]
Number of children (*n* = 2183)			
1 to 2	938	43	48 [45–51]
3 +	1245	57	52 [49–55]
Current use of birth control	1281	59	62 [59–65]
Believes public schools should teach sex education	1973	90	92 [90–93]
Believes adolescents get pregnant because of factors outside of their control (*n* = 2123)	1345	63	65 [62–68]

**Fig. 1:**
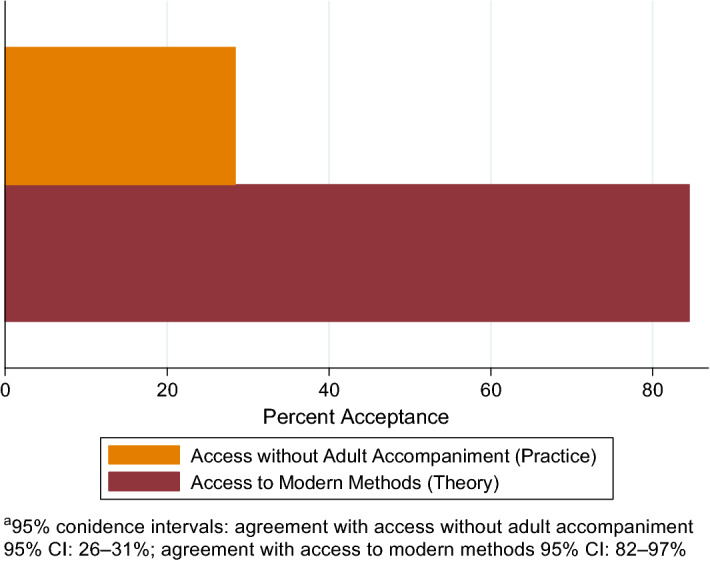
Agreement with adolescent access to contraception in theory and practice, Mexican Catholic parents, 2014 (*N* = 2, 186)

A large majority (85%; 95% CI: 82–87%) of our sample agreed that adolescents should have access to the full spectrum of modern contraceptive methods (access in theory; Fig. 1). A much smaller proportion (28%; 95% CI: 26–31%) believe that adolescents should be able to access contraceptive methods from a doctor without adult accompaniment (access in practice).

Everyone in the sample self-identified as Catholic as per the inclusion criteria, but only 14% (95% CI: 12–16%) reported being very Catholic (Table 2); half (51%; 95% CI: 48–54) of respondents said they were somewhat Catholic and the remainder not at all or a little Catholic. A large proportion (82%; 95% CI: 80–84%) agreed that a person who uses contraception can continue to be a good Catholic. A majority (84%; 95% CI: 82–85%) believe that the Church should permit Catholics to use contraception.

**Table 2:** Measures of Catholicism and agreement with adolescent access to contraception in theory and practice, Mexican Catholic parents, 2014 (n = 2186)

		Access in theory			Access in practice		
	Weighted %	Practice adolescents should have access to modern birth control methods (weighted %)			For doctors to prescribe birth control, adolescents should be able to go… (Weighted %)		
		Agree	Disagree	*p*-value*	Alone	Accompanied	*p*-value*
*Self-identified strength of Catholicism*				< .05			0.28
Very Catholic	14%	11%	3%		4%	10%	
Somewhat Catholic	51%	43%	8%		15%	36%	
Not at all/a little Catholic	35%	30%	5%		10%	25%	
*A person who uses birth control can continue to be a good Catholic*				< .001			<0 .001
Agree	82%	71%	11%		25%	57%	
Disagree	18%	13%	5%		4%	14%	
*God is someone who…*				0.77			0.13
Protects	36%	30%	6%		9%	27%	
Forgives, punishes, rewards, or imposes rules	64%	54%	10%		19%	45%	
*Respect is most important Catholic value*				0.6			0.97
Agree	32%	28%	4%		9%	23%	
Disagree	68%	57%	11%		19%	49%	
*The Catholic Church should permit Catholics to use birth control*				< 0.001			< 0.001
Agree	84%	74%	11%		25%	58%	
Disagree	16%	6%	10%		3%	13%	

*Chi2 *p*-value for differences between agreement or disagreement with adolescent access in theory and practice for each measure of Catholicism

Percents are rounded and may not always add to 100%

Table 2 presents the distribution of agreement or disagreement with adolescent access in theory and practice for each measure of Catholicism. Overall, weighted percentages are presented in the first column and the percent of agreement or disagreement with adolescent access among Catholic measure response items presented in subsequent columns. For example, 82% of the sample believe that a person who uses birth control can continue to be a good Catholic, broken down into 71% who agree and 11% who disagree that adolescents should have access to modern birth control (access in theory); the proportion of those agreeing to access in theory is significantly different by the response to the “good Catholic” measure (*p* < 0.001). In bivariate analysis, self-identified strength of Catholicism was significantly associated with support for access to contraception, with 43% of people who identify as somewhat Catholic and 30% of those identifying as not at all/a little Catholic supporting adolescent access to modern methods (*p* < 0.05, Table 2). Distribution of support for unaccompanied access to contraception in bivariate analysis was not significantly different by self-identified strength of Catholicism. People who believe that someone who uses contraception can continue being a good Catholic were significantly more likely to agree with both adolescent access to contraception (support in theory; *p* < 0.001) and no adult accompaniment to access contraception in bivariate analysis (support in practice; *p* < 0.001). Variables measuring idea of God and respect as the most important Catholic value were not significantly associated with either access in theory or practice. The variable measuring agreement that the Catholic Church should permit Catholics to use contraception was significantly associated with both of our outcomes in bivariate analysis; respondents who agreed that Catholics should be permitted to use contraception were significantly more likely to agree with adolescent access to modern methods in theory (*p* < 0.001) and unaccompanied access in practice (*p* < 0.001).

In multivariable models, self-identified strength of Catholicism was significantly and inversely associated with support for access to contraception in theory but not significantly associated with support for access in practice (Table 3). Belief that a person who uses contraception can continue to be a good Catholic was significantly associated with both outcomes, with respondents who believe that someone who uses contraception can continue to be a good Catholic having higher odds of supporting access to modern methods (aOR 2.4; 95% CI: 1.6–3.5) and unaccompanied access to contraception (aOR 1.5; 95% CI: 1.0–2.1). Support for public schools teaching sex education was not significantly associated with support for unaccompanied access to contraception (access in practice) when controlling for other factors, but was significantly associated with access to contraception in theory, (aOR 2.9; 95% CI: 1.8–4.5).

**Table 3:** Association of Catholicism and agreement with adolescent access to contraception in theory and practice, Mexican Catholic parents, 2014

	Access to modern methods (access in theory) *n* = 2103		Access without adult accompaniment (access in practice) *n* = 2105	
	aOR	95% CI	aOR	95% CI
*Self-identified strength of Catholicism (ref = very Catholic)*				
Somewhat Catholic	1.49	[0.987–2.237]	1.11	[0.749–1.641]
Not at all/a little Catholic	1.65*	[1.070–2.537]	1.07	[0.706–1.627]
*A person who uses birth control can continue to be a good Catholic (ref = disagree)*	2.36***	[1.614–3.455]	1.47*	[1.033–2.081]
*Public schools should teach sex education (ref = no)*	2.88***	[1.840–4.493]	1.2	[0.744–1.938]
*Adolescent pregnancy is due to factors within their control*	1.43*	[1.022–1.990]	1.38*	[1.048–1.805]
*Male sex of respondent (ref = female)*	1.35	[0.954–1.909]	0.87	[0.657–1.149]
*Age (ref = 18 to 30)*				
31 to 40	0.72	[0.432–1.200]	0.62*	[0.416–0.917]
41 to 50	0.99	[0.568–1.710]	0.8	[0.520–1.223]
51 +	0.79	[0.464–1.340]	0.87	[0.555–1.356]
*Education (ref = none or primary)*				
Secondary	0.66	[0.430–1.006]	1.2	[0.859–1.667]
High School or more	0.75	[0.450–1.244]	1.31	[0.884–1.936]
*Income, USD (ref = less than USD 145)*				
$145–434	0.81	[0.557–1.178]	1.11	[0.814–1.505]
$435–723	0.52*	[0.288–0.927]	0.93	[0.615–1.414]
724 +	0.63	[0.328–1.213]	1.12	[0.617–2.019]
*Mexico city residence (ref = outside)*	0.67	[0.412–1.098]	0.77	[0.486–1.210]
*Number of children (ref = 3 +)*				
1 to 2	1.36	[0.922–2.012]	1.27	[0.930–1.726]
*Current use of contraceptives*	1.39	[0.965–2.012]	1.18	[0.874–1.598]

*p-value < .05, **p-value < .01, ***p-value < .001

Age, education, income, Mexico City residence, use of contraception, and number of children were not consistently associated with our two outcomes. Parents who believe that adolescents get pregnant because of factors within their control had higher odds of agreeing with adolescent access to contraception in both theory (aOR 1.4; 95% CI: 1.0–2.0) and practice (aOR 1.4; 95% CI: 1.0–1.8) when compared to parents who think adolescents get pregnant because of factors outside their control.

## Discussion

Our results show that Mexican Catholic parents overwhelmingly (85%) support adolescent access to contraception overall (in theory), but that the operationalization of this support in practice (accessing contraception unaccompanied by an adult) is much lower (28%). Further, our results show strong support (92%) for sex education in schools and the Church to permit Catholics to use contraception (84%). These are important findings. Agreement that someone who uses contraception can continue being a good Catholic, and belief that adolescent pregnancy is due to factors within the adolescent’s control are both associated with support for access to contraception in theory and practice.

We show that the large majority of Catholic parents in Mexico believe that adolescents should have access to contraception. This echoes evidence from the United States, where adolescent access to contraception and especially sex education is widely supported, regardless of factors such as religion (Constantine et al. 2007; Barrett et al. 2014) and political affiliation (Kantor and Levitz 2017). Our findings also contribute to evidence that the individual Catholic opinions about sexual and reproductive health issues do not always align with the dictates of the Catholic hierarchy (Küng et al. 2018), and that Catholic people often find creative ways to reframe Catholic dictates to specific needs and desires (Hirsch 2008). Catholic opinion should not be seen as an unsurmountable barrier to implementing policies to expand adolescent access to modern contraceptive methods. In fact, the large majority of Mexican Catholic parents in our sample also believe the Catholic Church should permit Catholics to use contraception, showing support not only in terms of their own personal values but in terms of what the Church as an institution *should* do.

However, our results also show that Catholic parents have reservations about adolescents accessing contraception without an adult, what we labeled support in practice. Only 28% of our sample believe that adolescents should be able to receive contraception from a doctor without adult accompaniment. This may be due to a parent’s desire to accompany their children as a show of support or a perception of what it means to be a good parent in Mexico, and not necessarily opposition to their use of contraception. These findings are in line with previous work that describes parents’ concerns about sex education in Mexico arising primarily because of feeling left out or left behind, and not because of outright opposition (Pick et al. 2000). Literature has also documented a misalignment in Mexican-origin families in the US between when parents think their daughters should be autonomous and when those daughters consider themselves autonomous (Bácama-Colbert et al. 2012), suggesting that parents may think adolescents need accompaniment to access contraception, although adolescents themselves may not agree. More research is needed to distinguish between a parent’s desire to accompany a child as an expression of support, and a parent’s desire to prevent their child from accessing birth control without parental approval or consent. Adolescent pregnancy prevention interventions may benefit from approaches that involve both the adolescent and their parent. Specifically, framing support for the informed and autonomous decisions of adolescents over their sexual health and use of contraceptive methods as a demonstration of love and strong family relationships may help to encourage support for adolescent access without adult accompaniment, or may help build trust between parents and adolescents such that parental accompaniment is not a barrier to access.

Our findings also illustrate that often-used measures of Catholicism such as self-identified strength of Catholicism do not help explain opinion about adolescent access to contraception. Rather, it is the measure that asks about contraception and Catholicism together that is associated with both outcomes, suggesting that this measure better describes how respondents understand what it means to be Catholic and the behaviors expected of Catholics. This finding is supported in literature on abortion that also suggests that we lose nuance with broad measures of Catholicism (Küng et al. 2018). Previous work on the intersections of religiosity and contraception has urged researchers to “explore the interplay between how people interpret religious teachings as sources of opportunity and constraint” (Hirsch 2008). Our measure of belief that someone who uses contraception can continue being a good Catholic affords us the opportunity to operationalize how Catholic parents in Mexico reframe Catholic teachings to make certain behaviors acceptable considering their needs. Such findings can be used by advocates working to expand on-the-ground access to adolescent contraception in Mexico by designing targeted messages that work to expand understandings of what it means to be a “good” Catholic.

### Limitations

This study has a number of limitations. The survey was administered only to self-identified Mexican Catholics and may not be generalizable to Catholics in other countries, nor to other religious groups within Mexico. This is also a household survey and may skew respondents toward older Catholics who are more often in the home. However, the survey weights allow inference to the Catholic population. Data are from 2014. While some shifts away from the institution of religion have been observed in the country, the number of Mexican Catholics and the perspectives of the Catholic Church have remained largely stable over the past decade (Pew-Templeton 2020), with no major shifts in the public discourse around adolescent contraception. Therefore, we are comfortable that our analysis continues to reflect current realities. The survey was not administered to a comparison group of non-Catholics; we are unable to draw conclusions about differences between Mexican Catholics and non-Catholics. However, recruiting an appropriate comparison group in a culturally Catholic country like Mexico would likely prove difficult. Finally, state-level results would allow us to explore the diversity of Catholicism across Mexico’s 32 states, where some states are more conservative than others, but our survey does not permit state-level estimates.

## Conclusion

We found that Mexican Catholic parents in our sample overwhelmingly support adolescent access to modern contraception and sex education, in contrast to Catholic Church teachings. However, the majority of Mexican Catholic parents in our sample do not believe adolescents should be able to access contraception from a doctor without adult accompaniment, in contrast to national policy. More nuanced measures of Catholicism, such as agreement that a person who uses contraception can continue being a good Catholic, can help us frame messaging around and increase support for adolescent access to contraception.
